# ICBcomb: a comprehensive expression database for immune checkpoint blockade combination therapy

**DOI:** 10.1093/bib/bbad457

**Published:** 2023-12-12

**Authors:** Yun Xia, Yan Gao, Ming-Yu Liu, Lei Li, Wen Pan, Ling-Zi Mao, Zhongzheng Yang, Mei Yang, An-Yuan Guo

**Affiliations:** Hubei Bioinformatics and Molecular Imaging Key Laboratory, College of Life Science and Technology, Huazhong University of Science and Technology, Wuhan, 430074, China; Hubei Bioinformatics and Molecular Imaging Key Laboratory, College of Life Science and Technology, Huazhong University of Science and Technology, Wuhan, 430074, China; Hubei Bioinformatics and Molecular Imaging Key Laboratory, College of Life Science and Technology, Huazhong University of Science and Technology, Wuhan, 430074, China; Hubei Bioinformatics and Molecular Imaging Key Laboratory, College of Life Science and Technology, Huazhong University of Science and Technology, Wuhan, 430074, China; Hubei Bioinformatics and Molecular Imaging Key Laboratory, College of Life Science and Technology, Huazhong University of Science and Technology, Wuhan, 430074, China; Hubei Bioinformatics and Molecular Imaging Key Laboratory, College of Life Science and Technology, Huazhong University of Science and Technology, Wuhan, 430074, China; Wuhan Biobank Co., Ltd., Wuhan Institute of Biotechnology, Wuhan, 430000, China; Hubei Bioinformatics and Molecular Imaging Key Laboratory, College of Life Science and Technology, Huazhong University of Science and Technology, Wuhan, 430074, China; Hubei Bioinformatics and Molecular Imaging Key Laboratory, College of Life Science and Technology, Huazhong University of Science and Technology, Wuhan, 430074, China; Department of Thoracic Surgery, West China Biomedical Big Data Center, Med-X Center for Informatics, West China Hospital, Sichuan University, Chengdu, 610041, China

**Keywords:** database, immunotherapy, cancer, tumor immunology, bioinformatics, combination therapy

## Abstract

The success of immune checkpoint blockade (ICB) promotes the immunotherapy to be a new pillar in cancer treatment. However, the low response rate of the ICB therapy limits its application. To increase the response rate and enhance efficacy, the ICB combination therapy has emerged and its clinical trials are increasing. Nevertheless, the gene expression profile and its pattern of ICB combination were not comprehensively studied, which limits the understanding of the ICB combination therapy and the identification of new drugs. Here, we constructed ICBcomb (http://bioinfo.life.hust.edu.cn/ICBcomb/), a comprehensive database, by analyzing the human and mouse expression data of the ICB combination therapy and comparing them between groups treated with ICB, other drugs or their combinations. ICBcomb contains 1399 samples across 29 cancer types involving 52 drugs. It provides a user-friendly web interface for demonstrating the results of the available comparisons in the ICB combination therapy datasets with five functional modules: [1, 2] the ‘Dataset/Disease’ modules for browsing the expression, enrichment and comparison results in each dataset or disease; [3] the ‘Gene’ module for inputting a gene symbol and displaying its expression and comparison results across datasets/diseases; [4] the ‘Gene Set’ module for GSVA/GSEA enrichment analysis on the built-in gene sets and the user-input gene sets in different comparisons; [5] the ‘Immune Cell’ module for immune cell infiltration comparison between different groups by immune cell abundance analysis. The ICBcomb database provides the first resource for gene expression profile and comparison in ICB combination therapy, which may provide clues for discovering the mechanism of effective combination strategies and new combinatory drugs.

## INTRODUCTION

Since the concept of immune cells eliminating cancer cells [1] was first proposed, cancer immunotherapy has been a research hotspot for decades. After the success of the PD-1/PD-L1 [2] blockade and the CTLA-4 [3] blockade in animal models for cancer treatment, the immune checkpoint blockade (ICB) therapy for cancer has gained significant attention. The objective of ICB is to eliminate inhibitory pathways that impede the effectiveness of cytotoxic T lymphocyte responses [4]. Currently, the FDA has approved over 10 ICB drugs, including anti-PD-1, anti-PD-L1, anti-CTLA-4 and anti-LAG-3. As of 2022, over 85 tumor indications have been approved by the FDA for ICB [5]. However, the ICB monotherapy has obvious limitations, with its main drawback being a low treatment response rate. A significant proportion of patients do not derive benefits from the ICB therapy.

The ICB combination therapy is specifically designed to compensate for the limitations of ICB monotherapy [6]. ICB usually combines chemotherapy, targeted therapy and cancer vaccines [7, 8]. These combination therapies have demonstrated promising treatment outcomes in clinical trials [9]. In comparison with the ICB monotherapy, the ICB combination therapy has shown improvement in patient response rates, enhancement of anti-tumor effects and increased patient survival rates [10]. Besides the ongoing clinical trials, there is extensive preclinical research, particularly focusing on combining ICB with chemotherapy. The majority of these combination chemotherapy strategies involve the selection of drugs based on the established signaling pathways or the well-known anti-cancer targets [9]. Additionally, certain combination strategies are serendipitously discovered during experimental trials, particularly in the preclinical studies focusing on the combination of ICB and chemotherapy, which exhibit a lack of consistent macroscopic research patterns.

Currently, the proportion of clinical trials using the ICB monotherapy is decreasing, while the number of studies investigating the combination approaches is on the rise [11]. The importance of the ICB combination therapy is increasingly recognized by the researchers, leading to a surge in the related studies. Consequently, a substantial amount of sequencing data for the ICB combination therapy is dispersed throughout public databases, including NCBI Gene Expression Omnibus (GEO) and Sequence Read Archive (SRA). The integration of these data will enhance our understanding of the underlying mechanisms of the ICB combination therapy. Presently, ICBatlas [12] stands as the sole gene expression database related to the ICB treatment, and CanImmunother [13] is the database that provides a comprehensive knowledge of the experimentally validated cancer immunotherapies, their biomarkers, targets and control therapies. There is no dedicated database for the ICB combination therapy available. Hence, a comprehensive database integrating and analyzing the available expression data on the ICB combination therapy will provide clues for the transcriptomic features of the ICB combination treatment and will help screen the new combined candidates.

In this study, we developed a large-scale accessible data repository named ICBcomb. ICBcomb is an extensive resource that provides a comprehensive depiction of the transcriptomic characteristics of the ICB combination therapy. It contains the gene expression profiles and their analyses of 1399 preclinical samples (1288 mouse samples and 111 human samples), obtained from the published studies covering 29 different diseases. ICBcomb offers a user-friendly web interface, equipped with convenient browsing and searching functions that allow users to explore datasets, diseases, genes, pathways, user-input gene sets and immune cell types. To the best of our knowledge, ICBcomb is the first resource that provides the gene expression profiles in the ICB combination therapy and offers valuable insights for discovering new drugs for the ICB combination. The ICBcomb database is freely accessible at http://bioinfo.life.hust.edu.cn/ICBcomb/.

## METHODS

### Data collection and processing

We collected expression data related to ICB combination therapy from NCBI GEO, NCBI SRA and ArrayExpress by searching keywords ‘ICB/immune checkpoint inhibitor (ICI) combination therapy’ or ‘PD-1/PD-L1/CTLA-4/ anti-PD-1/ anti-PD-L1/anti-CTLA-4/combination therapy’ or ‘combination immunotherapy’. For ICBcomb, the collected datasets (Supplementary Table S1) must cover at least one non-ICB drug (synergistic drug) in combination with the ICB therapy and provide bulk RNA-seq data for at least one of the following five groups: ICB drug group versus Control group; Synergistic drug group versus Control group; Combination group versus Control group; Combination group versus ICB drug group and Combination group versus Synergistic drug group.

**Figure 1:**
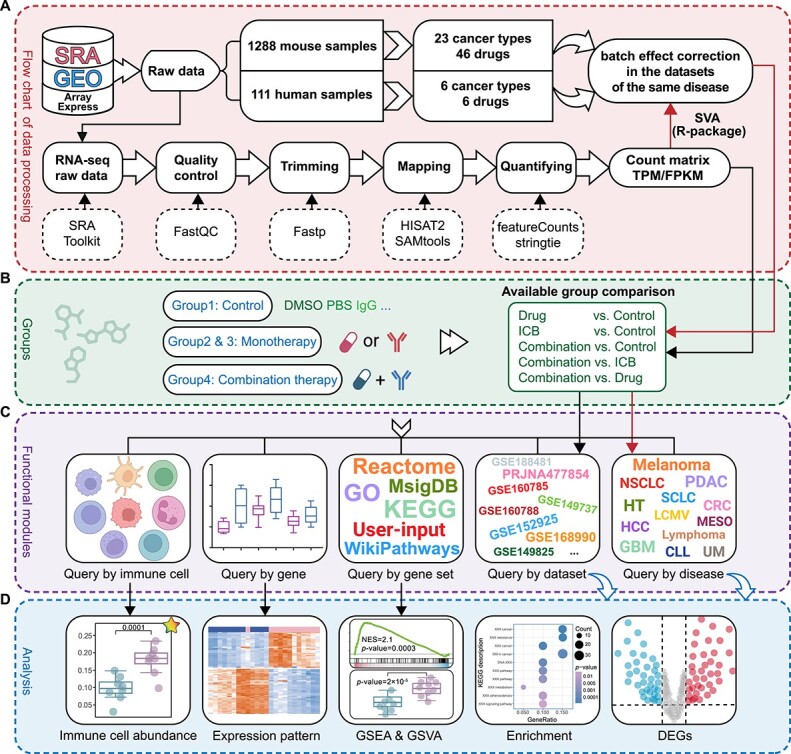
**Overview of the workflow for data processing and analysis.** (**A**) Overview of the data processing workflow of ICBcomb. (**B**) Grouping strategy diagram. (**C**) ICBcomb provides five functional modules, including browsing or searching by disease, dataset, gene, gene set and immune cell type. (**D**) The several key features provided by ICBcomb, including the identification of DEGs between available ICB combination groups, visualization of DEGs expression patterns through heatmaps, GO/the KEGG enrichment analysis of DEGs, GSEA, GSVA enrichment analysis and immune cell abundance analysis between groups.

We downloaded raw sequencing reads (if available) or other available formats (raw count, TPM, FPKM, etc.) for each dataset. Furthermore, experimental information for samples, including disease type, ICB treatment and drug dosage, was collected from the original article. For the raw sequencing data, SRA Toolkit (version 3.0.0) was used to download and convert the format of sra to fastq. FastQC (version 0.11.9) was used for data quality control (QC), and Fastp [14] (version 0.23.1) was employed for adapter sequence removal and trimming to obtain high-quality clean reads. Clean reads were mapped to the human reference genome GRCh38 or the mouse reference genome GRCm39 by HISAT2 [15] (version 2.2.1). SAMtools [16] (version 1.16) was used to convert the ‘.sam’ file into a ‘.bam’ file. StringTie [17] (version 2.2.1) was used to estimate the abundance of transcripts for each sample. FeatureCounts [18] (version 2.0.3) was used to calculate gene expression and get the raw counts. The parameter details of our bulk RNA-seq workflow were uploaded to GitHub (https://github.com/cloudsummer/ICBcomb). SVA [19] (R-package) was used to correct the batch effect. We screened studies for the ICB combination therapy and manually reviewed the study design to label the samples into four groups: Control group (not treated with ICB or its synergistic drug), Drug group (only treated with synergistic drugs), ICB group (only treated with ICB) and Combination group (treated with both ICB and its synergistic drug). Figure 1 provides an overview of the data processing and analysis.

### Differential expression analysis and functional enrichment analysis

The differentially expressed genes (DEGs) of the available comparisons (Drug group versus Control group, ICB group versus Control group, Combination group versus Control group, Combination group versus ICB group and Combination group versus Drug group, Figure. 1B) were calculated by DESeq2 [20]. For each dataset, after obtaining the reads matrix, we performed a minimal pre-filtering to keep only rows (each row represents the reads of a gene in different samples) that have at least 10 reads total. The DEGs from the raw count of RNA-seq were calculated by DESeq2 with the thresholds (|log2FoldChange| < log_2_(1.5) and adjust *P*-value <0.05).

We conducted pathway analysis for DEGs via Gene Ontology (GO) and Kyoto Encyclopedia of Genes and Genomes (KEGG) [21] pathways utilizing clusterProfiler [22] (R-package, version 4.8.1). Terms with FDR <0.05 and count >5 were considered as significantly functional enriched pathways. We used Gene Set Enrichment Analysis (GSEA) [23] to identify the gene expression signatures that were significantly altered between the specific groups. Besides, to increase the power of detecting subtle changes in pathway activity between groups, we performed Gene Set Variation Analysis (GSVA) [24] (GSVA, R-package, version 1.48.0) to calculate the gene set enrichment score per sample. GSEA and GSVA analyses were used to explore the changes in the overall expression patterns of the gene set or pathway as follows: [1] Human: human hallmark gene sets of Molecular Signature Database (version 7.4, MSigDB, http://www.gsea-msigdb.org/gsea/index.jsp), Reactome and WikiPathways of Canonical pathways (CP), C6 subsets, C7 subsets and C8 subsets of the MSigDB; [2] Mouse: mouse hallmark gene sets of MSigDB, canonical pathways of Reactome and WikiPathways, MPT gene sets (92 gene sets mined from the Mammalian Phenotype Ontology database corresponding to tumor-specific ontology terms) and M8 subsets of the MSigDB. FDR <0.05 and normalized enrichment score (|NES| > 1) indicated significant gene enrichment (GSEA). Mann–Whitney U test was used to calculate the *P*-value in the GSVA enrichment analysis, and *P*-value <0.05 indicated significant results (GSVA).

### Tumor infiltration analysis by ImmuCellAI-mouse and mMCP-counter

We performed immune cell abundance analysis in tumor tissue sources. We adopted the Immune Cell Abundance Identifier (ImmuCellAI-mouse) [25] in mouse datasets for the estimation of the abundance of 36 immune cell types: B cells (including subtypes of B1, follicular B, germinal center B, marginal zone B, memory B and plasma B cells), T cells (including subtypes of CD4^+^ T, CD8^+^ T, NKT, and γσ T cells), myeloid cells (four subtypes of macrophages, DCs, monocytes and granulocytes) and NK cells. Furthermore, ImmuCellAI-mouse [25] evaluated the cellular subtypes closely associated with CD8^+^ and CD4^+^ T cells, which are crucial in tumor immunity, including CD4^+^ naïve, CD4^+^ memory, Treg, T helper, CD8^+^ naïve, CD8^+^ central memory, CD8^+^ effector memory, cytotoxic and exhausted CD8^+^ T cells. Additionally, we used mMCP-counter [26] for the assessment of the relative abundance of 12 distinct immune cell types (T cells, CD8^+^ T cells, NK cells, B-derived cells, memory B cells, monocytes/macrophages, monocytes, granulocytes, mast cells, eosinophils, neutrophils, basophils) and four stromal cell populations (vessels, lymphatics, endothelial cells and fibroblasts). We demonstrated the differences in immune cell abundance between groups using box plots and assessed the statistical significance between groups using the Mann–Whitney U test.

### Custom gene set search

This module provides the GSEA enrichment analysis based on specific organisms and user-input gene sets. After user submission, the interface will display results of the GSEA enrichment analysis in specific comparisons between different ICB combination therapy groups, including Drug versus Control, ICB versus Control, Combination versus Control, Combination versus ICB and Combination versus Drug.

### Construction of the database

ICBcomb was developed using the Python Flask-RESTful API framework (https://flask-restful.readthedocs.io/). The front-end pages were rendered and interacted with HTML, CSS, JavaScript (https://angularjs.org) and Vue2 (https://v2.cn.vuejs.org/). MongoDB (version 6.0.3, https://www.mongodb.com/) was used to organize and query the backend data. Charts were generated with Echarts and R scripts. Finally, the bioinformatics analyses were carried out by R. ICBcomb runs on the Apache HTTP Server (https://httpd.apache.org/).

## RESULTS

### Data summary of ICBcomb

The ICBcomb database includes expression data from both human and mouse samples. Currently, it contains 1288 mouse samples from 66 datasets (Statistical criteria: a dataset comprises research on one specific disease within a single species for one particular synergistic ICB drug) treated with PD-1/PD-L1, CTLA-4 and Tim3 blockade (Table 1). These mouse datasets include samples treated with ICB or its synergistic drug monotherapy, combination therapy of ICB with its synergistic drug and control samples. These samples cover 23 disease types, including breast cancer (BC), colorectal carcinoma (CRC), gastric cancer (GC), glioblastoma (GMB), hepatocellular carcinoma (HCC), lung cancer (LC), lymphocytic choriomeningitis virus infection (LCMV infection), lymphoma, melanoma, mesothelioma (MESO), pancreatic ductal adenocarcinoma (PDAC), hydrodynamic transfection of c-Myc and N-ras (HT), non-small cell lung cancer (NSCLC), head and neck squamous cell carcinoma (HNSCC), metastatic colorectal cancer (mCRC), triple-negative breast cancer (TNBC), bladder cancer (BLCA), small cell lung cancer (SCLC), Malignant peripheral nerve sheath tumors (MPNSTs), Neuroblastoma (NB), mice colorectal carcinoma (mCC); ovarian cancers (OV) and mesothelioma (MSTO). And the mouse datasets in ICBcomb involve 46 drugs, including Crizotinib, Cisplatin, Trametinib, Palbociclib, 5-Fluorouracil, RXDX-106, Celecoxib, Swainsonine, Rigosertib, IL-10-Fc, EPAT, Birinapant, anti-Cd40, TLR, ACT, IL-2, SD70, JQ1, Decitabine, TMAO, D18, ENMD, TAK, DSF, Dasatinib, Doxorubicin, PIIO-1, Aflibercept, anti-IL-17, Regorafenib, SN-38, poly-IC, BMS-595, YKL-5-124, Pemetrexed-Cisplatin, Palbociclib plus Mirdametinib, R788 (fostamatinib), Tretinoin, LGG, α-KG, PB-020, a sulfur acid-restricted diet (SARD), CXD101, Domatinostat and Poly (ADP-ribose) polymerase (PARP) inhibitors (PARPi). Additionally, the ICBcomb database contains 111 human samples from 10 datasets treated with PD-1/PD-L1 inhibitors across six disease types: chronic lymphocytic leukemia (CLL), HCC, lymphoma, melanoma, uveal melanoma (UM) and colorectal carcinoma (CRC) (Table 1). These human datasets involve six drugs, which are Entinostat, JQ1, anti-ILT3, IL-15, ascorbic acid and Avadomide.

**Table 1:** Summary of meta information of ICBcomb database

	Human	Mouse	Total
Datasets	10	66	76
Disease types	6	23	28
Samples	111	1288	1399
Control group	47	374	421
ICB group	9	276	285
Drug group	43	331	374
Combination group	12	307	319
Disease			
CLL	38	-	38
Melanoma	26	331	357
UM	24	-	24
HCC	6	12	18
Lymphoma	12	8	20
CRC	5	333	338
LC	-	150	150
MESO	-	74	74
PDAC	-	56	56
NSCLC	-	40	40
TNBC	-	40	40
LCMV Infection	-	30	30
HT	-	12	12
BC	-	12	12
GBM	-	9	9
SCLC	-	8	8
mCRC	-	16	16
GC	-	16	16
HNSCC	-	12	12
BLCA	-	10	10
MPNSTs	-	12	12
NB	-	6	6
AB1-HA (MSTO)	-	39	39
MC38 (mCC)	-	36	36
OV	-	26	26
Antibody			
anti-PD-1	86	964	1050
anti-PD-L1	20	100	120
anti-CTLA-4	-	10	10
anti-PD-1 + anti-CTLA-4	5	58	63
anti-PD-L1 + anti-CTLA-4	-	139	139
anti-PD-1 + anti-Tim3	-	17	17

Abbreviations: CLL, Chronic Lymphocytic Leukemia; UM, Uveal Melanoma; CRC, Colorectal Carcinoma; MESO, Mesothelioma; PDAC, Pancreatic Ductal Adenocarcinoma; NSCLC, Non-Small Cell Lung Cancer; SCLC, Small Cell Lung Cancer; LCMV Infection, Lymphocytic Choriomeningitis Virus Infection; GBM, Glioblastoma; LC, Lung Cancer; GC, Gastric Cancer; BC, Breast Cancer; HT, Hydrodynamic Transfection of c-Myc and N-ras; HCC, Hepatocellular Carcinoma; mCRC, Metastatic Colorectal Cancer; TNBC, Triple-Negative Breast Cancer; BLCA, Bladder Cancer; HNSCC, Head and Neck Squamous Cell Carcinoma; MPNSTs, Malignant Peripheral Nerve Sheath Tumors; NB, Neuroblastoma; MC38 (mCC), MC38 (murine colorectal carcinoma); OV, ovarian cancers; AB1-HA (MSTO), AB1-HA (mesothelioma)

### Modules and functions

In ICBcomb, there are five functional modules, representing five different ways of querying data, including Dataset, Disease, Gene, Gene Set and Immune Cell modules (Figure 1C). Each module provides a download function. We analyzed the expression data by comparing different groups, which are ICB drug, synergistic drug, combination (ICB + synergistic drug) and control groups. The five modules were described in detail in the following:

### Dataset, disease and gene modules

**Figure 2:**
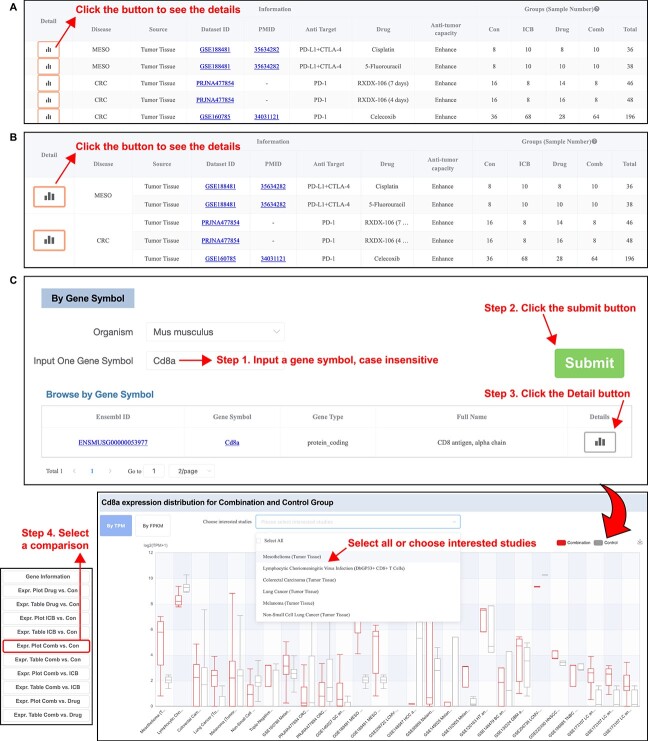
Dataset, disease and gene module in ICBcomb. (**A**) Dataset module. Con: Control group (not treated with ICB drugs or non-ICB drugs), ICB: ICB group (only treated with ICB drugs), Drug: Drug group (only treated with non-ICB drugs, usually synergistic drugs), Comb: Combination group (treated with both ICB drugs and non-ICB drugs) (**B**) Disease module (C) Gene module.

The ‘Dataset’ module summarizes the disease type, anti-target (ICB), combinatory drug and sample information for each dataset. The ‘Disease’ module aggregates samples belonging to the same disease and corrects the batch effect of samples from different datasets of the same disease. Users can select the interested dataset or disease in the table to browse the detailed results, such as gene expression, pathway enrichment and immune cell abundance. Dataset and Disease modules include analyses of DEGs, GO/KEGG enrichment analysis of DEGs, GSEA, GSVA enrichment analysis and immune cell abundance by comparing different treatment groups (Figure 2A, 2B). The ‘Gene’ module provides a way to query a gene and presents the results of expression and enrichment between available group comparisons (Figure 2C).

### Gene set module

**Figure 3:**
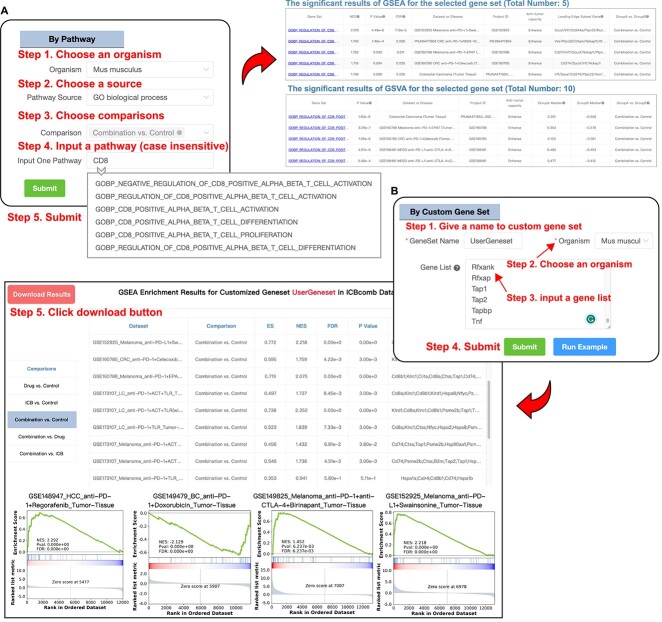
Gene Set module in ICBcomb. (**A**) Step-by-step instructions for the ‘By Pathway’ function. ‘By Pathway’ is one of functions in the ‘Gene Set’ module (**B**) Figure illustrating the step-by-step instructions for the ‘By Custom Gene Set’ function. ‘By Custom Gene Set’ is another function in the ‘Gene Set’ module.

The ‘Gene set’ module aims to analyze the gene expression enrichment pattern of a specific pathway (Figure 3A) or custom gene set (Figure 3B) in different ICB combination therapy groups. This module helps users examine the performance of the user-input gene set in different ICB groups, thereby validating whether the overall expression of the user-input gene set is positively/negatively correlated with those present in ICB combination therapy datasets of ICBcomb. It provides great convenience for users to find biomarkers in the ICB combination therapy. The ‘Gene set’ module consists of two parts: the ‘By Pathway’ sub-module and the ‘By Custom Gene Set’ sub-module (Figure 3A, B).

The ‘By Pathway’ sub-module presents the GSEA and GSVA enrichment results based on pathways (gene sets) from Reactome, WikiPathways, GO, the KEGG and MSigDB in a single dataset or multiple datasets across the same disease, only showing significant results (*P*-value <0.05). The GSEA and GSVA enrichment results are based on specific comparisons between available ICB combination therapy groups (Figure 1B).

The ‘By Custom Gene Set’ sub-module provides the GSEA enrichment analysis based on user-input gene sets. After user submission, it will display results of the GSEA enrichment analysis in specific comparisons between available ICB combination therapy groups. After performing GSEA enrichment analysis, users can download these results in the form of tables and plots (Figure 3B). The function of these two sub-modules of analysis serves to enhance the users’ understanding of ICB combination therapy on their preferred pathways or gene sets.

### Immune cell module

**Figure 4:**
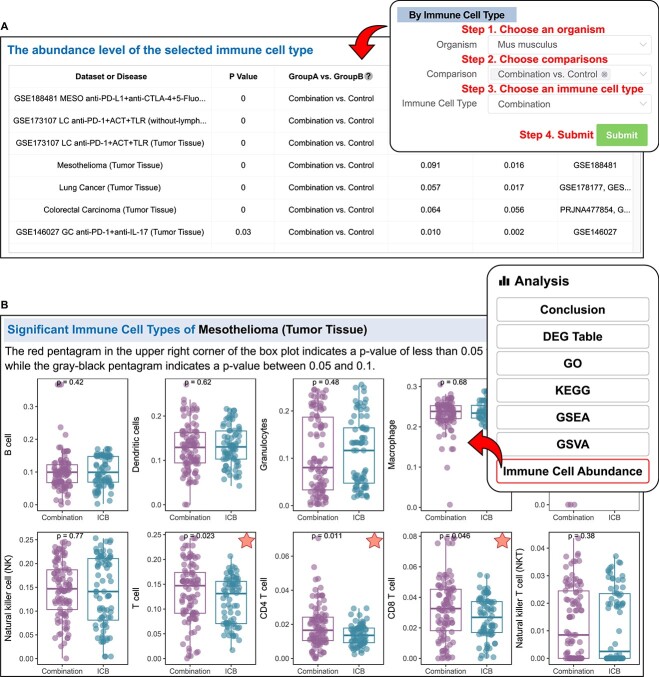
Immune Cell module in ICBcomb. (**A**) Figure illustrating the step-by-step instructions for the ‘Immune Cell’ module. (**B**) One result of the ‘Immune Cell Abundance’ function of the ‘Datasets/Disease’ module. The red star means the significant comparison (*P*-value <0.05).

The ‘Immune Cell’ module allows users to compare the immune cell infiltration in specific ICB combination therapy groups across diverse datasets by choosing an immune cell type (Figure 4A). If users are interested in the immune infiltration of a specific dataset or disease, they can query it using the dataset or disease module. In each available comparison, the results of ‘Immune Cell Abundance’ are at the bottom of the section (Figure 4B).

## CASE STUDY

### The gene expression pattern exploration and mechanism inference in the ICB combination therapy datasets of NSCLC

Immunotherapy is emerging as a crucial approach in the treatment of NSCLC [27]. Ongoing clinical trials are exploring ICB combination therapy approaches involving chemotherapy and targeted drugs. Additionally, some studies are investigating novel drugs that can augment the therapeutic efficacy of ICB in NSCLC. However, current research lacks cross-dataset analysis of multiple ICB combination therapy datasets in NSCLC, which is essential to explore the patterns of gene expression and underlying mechanisms. Currently, our ICBcomb can address this.

**Figure 5:**
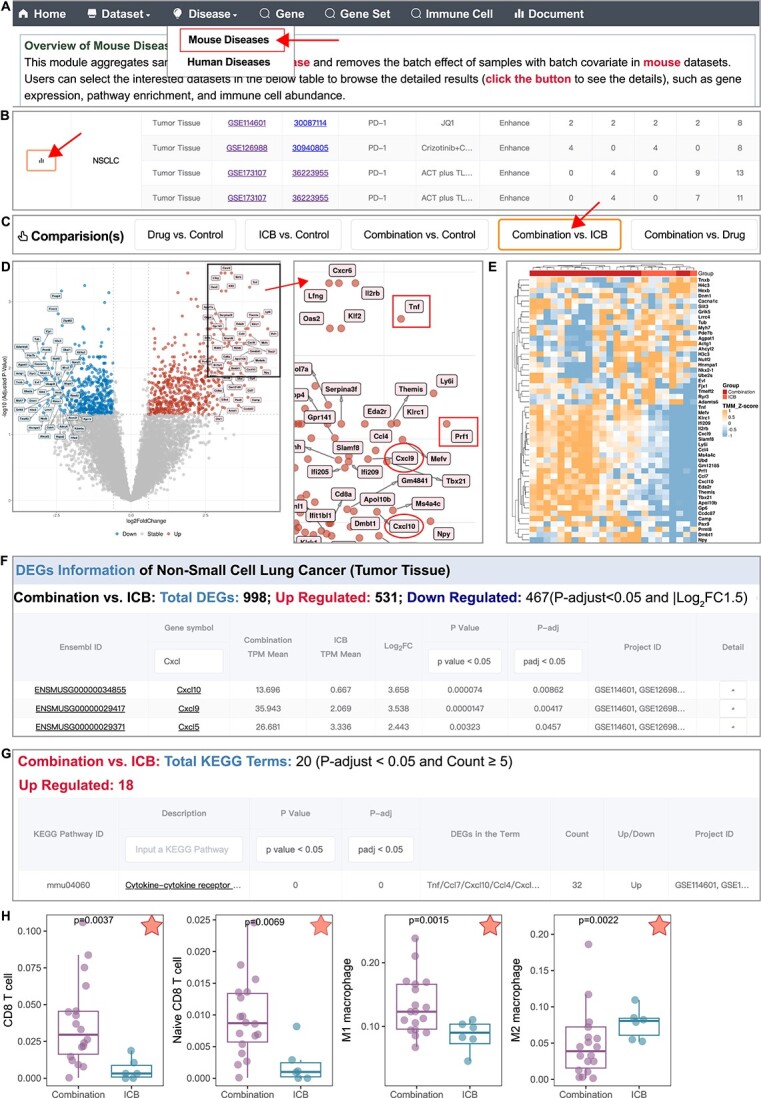
**A case study of ICBcomb to explore the mechanism of ICB combination therapy in NSCLC. (A)** Functional module selection. (**B**) Part of the snapshot of the ‘Disease’ page with NSCLC datasets detail. (**C**) Available comparisons of NSCLC. (**D**) DEGs volcano plot of ‘Combination versus ICB’ in NSCLC. (**E**) DEGs heatmap of ‘Combination versus ICB’ in NSCLC. (**F**) Snapshot of DEGs search interface of ‘Combination versus ICB’ in NSCLC. (**G**) A Snapshot of significant results displays the interface of KEGG Enrichment Analysis. (**H**) Immune cell abundance comparison in the combination therapy group and the ICB monotherapy group of CD8^+^ T cells, naïve CD8^+^ T cells, M1 macrophage and M2 macrophage. The red star in the upper right corner represents *P*-value <0.05. *P*-value by Mann–Whitney U-test.

Through ICBcomb, users can easily obtain the results with a few simple mouse clicks. First, users click ‘Disease’ at the top menu and choose the ‘Mouse Diseases’ module (Figure 5A). Then, click the first column on the line of NSCLC (Figure 5B). Next, select the ‘Combination versus ICB’ panel at the top of the page (Figure 5C) to explore the gene expression changes of combination therapy compared with the ICB monotherapy. This will redirect to a detailed results page comparing these two groups. We can easily enlarge the volcano plot through clicking it to visually observe DEGs (Figure 5D). Among the upregulated DEGs (highlighted in red), we can see ‘Tnf’ and ‘Prf1’, which are molecules highly correlated with cytotoxicity [28, 29]. Moreover, the presence of Cxcl10 and Cxcl9 in upregulation genes can be observed, which are considered as key molecules closely associated with T cell infiltration in immunotherapy [30, 31]. Heatmaps can be utilized for visualizing the expression patterns of the most significant DEGs in each individual sample (Figure 5E). To identify the additional upregulated DEGs in the Cxcl chemokine family, we simply input ‘Cxcl’ under the DEGs section, which will result in Cxcl10, Cxcl9 and Cxcl5 as DEGs (Figure 5F). Among the upregulated DEGs, the most significantly enriched pathway in the KEGG enrichment analysis is ‘Cytokine–cytokine receptor interaction’ (Figure 5G), and ‘Chemokine signaling pathway’ is ranked fifth in the plot of KEGG enrichment. DEGs analysis and KEGG results suggested that significant upregulation of numerous cytokines and chemokines were associated with enhancing the anti-tumor effect of ICB combination therapy.

In the analysis of immune cell abundance, with the ‘red star’ marker in the upper right corner, we can quickly identify several significant results, including CD8^+^ T cells, naïve CD8^+^ T cells, M1 macrophages and M2 macrophages (Figure 5H). The results indicate that the combination therapy group, compared with using the ICB treatment alone, exhibits a significant increase in CD8^+^ T cells and M1 macrophage abundance, but a reduction in M2 macrophage abundance. These results were consistent with the reports that M1 macrophage enhances the function of cytotoxic T cells and anti-tumor immunity [32], while M2 macrophage promotes tumor progression [33]. Taking into account the previous discovery of Cxcl9 and Cxcl10 as upregulated DEGs and the pathway enrichment results, we inferred that in NSCLC treated with ICB combination therapy, the production of Cxcl9 and Cxcl10 in the tumor microenvironment promotes the infiltration of CD8^+^ T cells [34] and helps in the progression of ‘hot’ tumors [35]. Furthermore, we inferred that in NSCLC, ICB combination therapy polarizes macrophages toward the M1 phenotype while inhibits M2 macrophage polarization.

## DISCUSSION

Currently, for clinical cancer treatment based on ICB, the strategy is to develop the ICB combination therapies with the tumor-targeted chemotherapy drugs or radiation therapy [8]. Although there are many sequencing data scattered in different public databases, there is no specific database for the ICB combination therapy. A comprehensive database exploring the gene expression pattern and pathway for the ICB combination therapy will help to understand the mechanism and identify new drugs. Thus, we constructed ICBcomb, which is the first molecular database for the ICB combination therapy and provides a comprehensive analysis of gene expression with five functional modules of Datasets, Disease, Gene, Gene set and Immune cell. Users have the option to choose suitable functional modules to query the analysis results that are relevant to their specific research objectives. Thus, they can explore the underlying mechanisms and inspire improvement in experimental designs. Through ICBcomb, in the case study on the ICB combination therapy in NSCLC, we deduced that the ICB combination therapy promotes the polarization of M2 macrophages toward the M1 type, leading to an augmented infiltration of the CD8^+^ T cells into tumor tissues and enhancing the therapeutic effect of anti-PD-1. Evidence from liver cancer research [36] of similar mechanisms can support our inference. All these results solidly confirmed the practicality and convenience of ICBcomb for the immunotherapy researchers.

The analysis results provided by ICBcomb were usually interesting to researchers. Experimental validation of mechanisms is typically performed using these results as a guide. The results provided by ICBcomb, including the analysis presented in the aforementioned case study, can serve as a clue for the development of the ICB combination therapy and facilitate the progress of immunotherapy. In addition, ICBcomb has a user-friendly interface and can be accessed freely by the academic users without registration. As we enter the era of the ICB combination therapy, studies on the application of the ICB combination treatment are steadily growing. In the future, ICBcomb will integrate additional datasets encompassing a wider range of combination treatment types. Consequently, ICBcomb has the potential to become an indispensable resource for the field of immunotherapy.

**Key Points**

- ICBcomb is the first gene expression database for the ICB combination therapy (ICB drugs + non-ICB drugs). The data in ICBcomb contain 52 different ICB combination drugs and a total of 1399 samples, spanning 29 diseases in both human and mouse models.
- The database features a user-friendly interface with modules of Dataset, Disease, Gene, Gene Set and Immune Cell.
- The Case Study section illustrates how the database can assist researchers in gaining novel insights.
- The gene set module allows users to explore the activated or the inhibited common biological processes or their input gene list across various datasets, which is crucial for the development of the novel ICB combination therapy drugs.

## Supplementary Material

Supplementary_Table_S1_bbad457

## Data Availability

All data are available online http://bioinfo.life.hust.edu.cn/ICBcomb.

## Author Biographies

**Yun Xia** is a PhD student at Huazhong University of Science and Technology, China, working on tumor bioinformatics.

**Yan Gao** is a master student at Huazhong University of Science and Technology, China, working on database construction.

**Ming-Yu Liu** is an undergraduate student at Huazhong University of Science and Technology, China, working on bioinformatics.

**Lei Li** is an undergraduate studentat Huazhong University of Science and Technology, China, working on bioinformatics.

**Wen Pan** PhD is a student at Huazhong University of Science and Technology, China, working on tumor bioinformatics.

**Ling-Zi Mao** is a master student at Huazhong University of Science and Technology, China, working on tumor bioinformatics.

**Zhongzheng Yang** an employee at Wuhan Biobank Co., Ltd., Wuhan Institute of Biotechnology, China, working on biological database.

**Mei Yang** is a PhD student at Huazhong University of Science and Technology, China, working on tumor bioinformatics.

**An-Yuan Guo** is a professor at Huazhong University of Science and Technology and West China Hospital, Sichuan University, China, working on tumor bioinformatics.
